# A naturalistic fMRI dataset in response to public speaking

**DOI:** 10.1038/s41597-025-05017-5

**Published:** 2025-04-19

**Authors:** Bolong Wang, Xuanxuan Zhang, Linmiao Zhang, Xiang-Zhen Kong

**Affiliations:** 1 Department of Psychology and Behavioral Sciences, Zhejiang University, Hangzhou, China; 2 The State Key Lab of Brain-Machine Intelligence, Zhejiang University, Hangzhou, China; 3 Department of Psychiatry of Sir Run Run Shaw Hospital, Zhejiang University School of Medicine, Hangzhou, China

**Keywords:** Language, Social neuroscience

## Abstract

Public speaking serves as a powerful tool for informing, inspiring, persuading, motivating, or entertaining an audience. While some speeches effectively engage audience and disseminate knowledge, others fail to resonate. This dataset presents functional magnetic resonance imaging (fMRI) data from 31 participants (14 females; age: 22.29 ± 2.84 years) who viewed two informative speeches with varying effectiveness, selected from YiXi talks (similar to TED Talks), and matched in length and topic. A total of 22 participants (10 females; age: 22.64 ± 2.77 years) who completed the full task were included in the validation analyses. A comprehensive validation process, involving behavioral data analysis and head motion assessment, confirmed the quality of the fMRI dataset. While previous analyses have used inter-subject correlation to examine neural synchronization during the reception of informative public speaking, this dataset can be utilized for a variety of analyses to further elucidate the neural mechanisms underlying audience engagement and effective communication.

## Background & Summary

Naturalistic paradigms are gaining momentum in cognitive neuroscience due to their ecological validity compared to traditional experimental tasks^1–3^. Advances in methodologies for analyzing interpersonal neural synchronization, such as inter-subject correlation (ISC)^4,5^, have enabled researchers to investigate the intricate dynamics of the human cognition and behavior in real-life contexts^6–9^. Previous studies using these paradigms have examined brain activity in response to various naturalistic stimuli reflecting everyday experiences, including movies, spoken dialogue, narratives, text, and music^10–17^. However, public speaking remains an underexplored yet crucial stimulus. As a common form of communication, public speaking is a powerful tool for informing, inspiring, persuading, motivating, and entertaining audiences^18^. While some speeches successfully engage and convey insights, others fail to resonate. Exploring how public speaking engages audiences can yield valuable insights into effective communication and education sciences.

Different types of public speaking—persuasive, entertaining, and informative—engage listeners’ brains in distinct ways. Persuasive and entertaining speeches often rely on rhetorical techniques, storytelling, and emotional appeals to captivate audiences. Research has demonstrated increased neural synchronization in response to rhetorically powerful political speeches^19^, compelling narratives^12,20^, and effective persuasive health messages^21,22^. In contrast, informative public speaking, like teaching lectures designed to convey complex and abstract knowledge from an expert to a novice^23^, demands precise, concise communication to maximize comprehension and retention. Despite the unique challenges of this communication form, the neural underpinnings of audience engagement during informative public talks remain underexplored, with no publicly available dataset addressing brain responses to public speaking.

In this paper, we present a naturalistic functional magnetic resonance imaging (fMRI) dataset collected to explore the neurobiological basis of audience responses to informative public speaking^24^. This dataset includes fMRI images from 31 participants (14 females; age: 22.29 ± 2.84 years) during two runs, as they watched two informative speech videos matched in length (approximately 24 minutes each) and topic (art and design) but differing in effectiveness based on audience engagement ratings. Additionally, T1-weighted structural images are included in the dataset.

While it was primarily designed for ISC analysis^4,5^, the data is well-suited to answering a broad range of research questions related to public speaking and prolonged brain activity dynamics. Future work could expand its scope by incorporating behavioral or physiological measures or comparing informative public speaking with teaching or other communicative contexts. Such efforts could deepen our understanding of the neural mechanisms involved in audience engagement and knowledge transfer, contributing to a more comprehensive understanding of effective communication.

## Methods

### Participants

Forty-eight young adult participants (20 females; age: 22.07 ± 2.35 years) with normal hearing and normal or corrected-to-normal vision were recruited. Seventeen of them (9 females; age: 21.76 ± 1.75 years) completed the speech watching and rating task for speech video stimuli preparation. Thirty-one right-handed participants (14 females; age: 22.29 ± 2.84 years) were recruited to complete the fMRI experiment, among which one participant was excluded for falling asleep during scanning, five for silent presentation of stimuli, and three for incomplete data due to technical issues or late arrival, resulting in 22 participants (10 females; age: 22.64 ± 2.77 years) in the fMRI data analysis. All participants were fluent Chinese speakers and provided written informed consent. They were recruited through online platforms to participate in both behavioral and fMRI experiments. All procedures were approved by the Institutional Review Board of Zhejiang University (No. 2023053). Prior to the experiments, written informed consent was obtained from each participant, explicitly authorizing the use of their anonymized data for research publication and open-access sharing.

### Speech video stimuli preparation

**Fig. 1:**
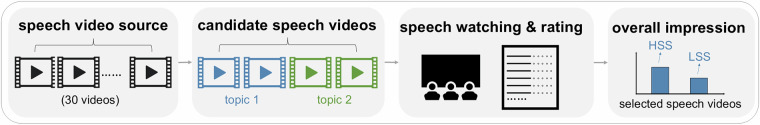
Schematic of fMRI stimuli preparation. A three-step approach was employed to determine the two speech videos with differential engagement levels selected from a larger set of candidates.

**Fig. 2:**
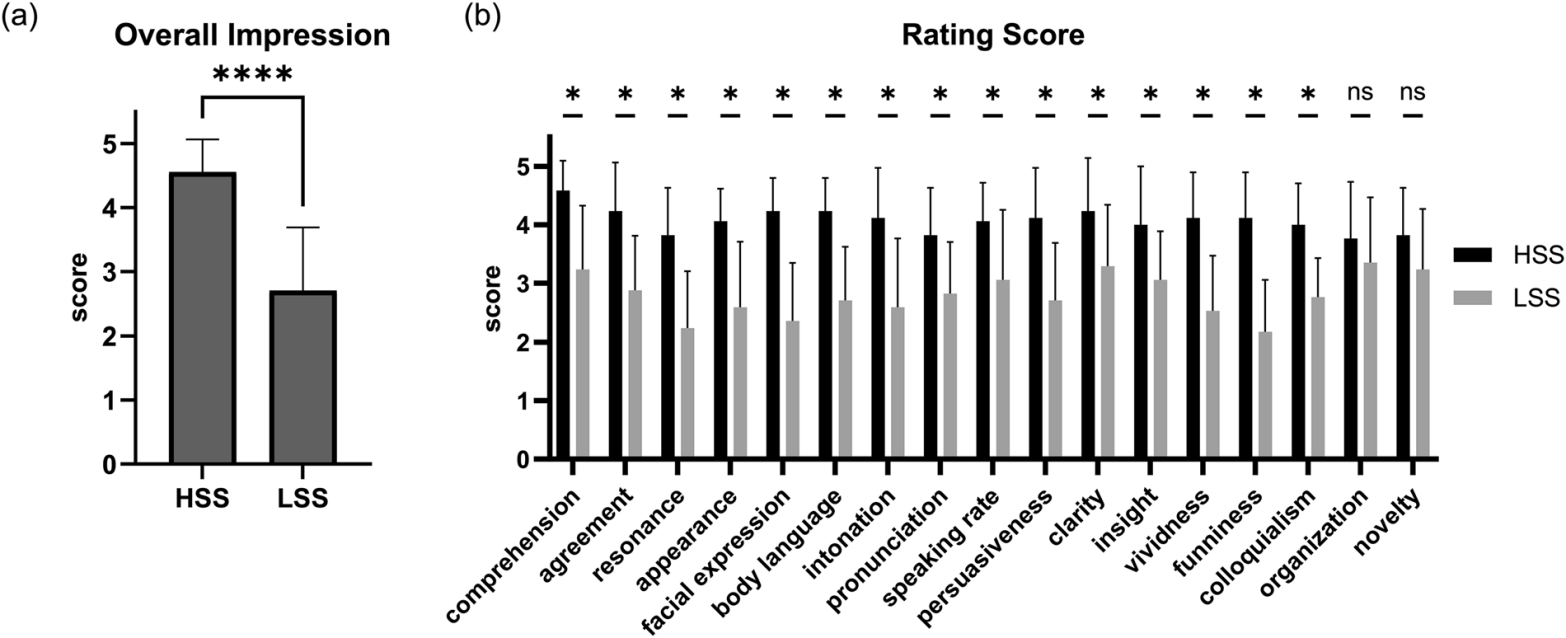
Rating results of the speech videos. (**a**) Overall impression ratings of the HSS and LSS. The results revealed a significant difference in scores between HSS and LSS (paired t-test, *t*(16) = 6.080, **** *p* < 0.0001). **(b)** Ratings of HSS and LSS on the 17 sub-items of the evaluation questionnaire. The results showed significant differences in scores between these two speeches on most sub-items (paired t-test, * *p* < 0.05).

To determine the speech video stimuli for the fMRI experiment, we adopted a three-step approach (see Fig. 1). Firstly, an initial group of speech videos (n = 30, see Table 1 and Table S1) was selected from YiXi talks (https://yixi.tv/, which is similar to TED). These speeches differ in length (28.64 ± 4.57 mins) and cover various topics (e.g., art and design, literature, science, etc.). All speech videos are subtitled. Then, we selected two pairs of candidate speech videos from the initial list based on the following criteria: (1) the two speeches in each pair differ significantly in terms of their level of engagement, (2) they share similar themes, and (3) they have similar lengths, with a difference of no more than 2 minutes between them. Finally, a group of participants (N = 17, determined by availability) were recruited for an independent assessment of these candidate speeches. Specifically, each participant was instructed to watch these four speech videos in turn and answer a set of 18 questions about their engagement with each video. The questions consisted of an overall rating of an individual’s engagement, along with multiple detailed dimensions of a speech: comprehension, agreement of viewpoints, emotional resonance, appearance, facial expression, body language, intonation, pronunciation, speaking rate, persuasiveness, clarity, organization, insight, novelty, vividness, funniness, and colloquialism (see Table 2). A higher score on each question indicates higher engagement. Then, two speech videos with larger differences in the overall impression were finally selected (Table 1), and the detailed questions were used to validate their differences in engagement (see Fig. 2). The length of the higher-scoring speech (HSS) was 24'03'' and the lower-scoring speech (LSS) was 23'38''. These durations were slightly shorter than the original videos available on the official website, as the introductory advertisement segments (10 seconds for HSS and 50 seconds for LSS) were removed. Both speeches were about topics of art and design. These obtained speech videos were used as stimuli in the subsequent fMRI experiment.

**Table 1:** Selected speech videos and detailed information.

Title	Time	Topic	Duration	Link
What’s wrong with our design	2018.04.15	Design	24'13''	https://www.yixi.tv/#/speech/detail?id=646
Bigger	2016.10.30	Design	24'28''	https://www.yixi.tv/#/speech/detail?id=303

**Table 2:** Speech Rating Questionnaire.

Number	Question
**Q1**	Your overall impression of this speech is very positive.
**Q2**	You have a high level of comprehension of this speech.
**Q3**	You agree with the viewpoints of this speech.
**Q4**	You have a high level of emotional resonance with this speech.
**Q5**	The speaker’s appearance is excellent.
**Q6**	The speaker’s facial expression is appropriate.
**Q7**	The speaker’s body language is appropriate.
**Q8**	The speaker’s intonation is appropriate.
**Q9**	The speaker’s pronunciation is excellent.
**Q10**	The speaker’s speaking rate is appropriate.
**Q11**	The speech’s content is persuasive.
**Q12**	The speech’s content is clear.
**Q13**	The speech’s content is well-organized.
**Q14**	The speech’s content is insightful.
**Q15**	The speech’s content is novel in its viewpoints.
**Q16**	The speech’s content is vivid and imaginative.
**Q17**	The speech’s content is engaging.
**Q18**	The speech’s colloquial style is appropriate.

**Note**: The questionnaire consisted of 18 questions, each rated on a five-point Likert scale. Participants were instructed to evaluate each question based on their agreement, using the following options: “Strongly Disagree,” “Disagree,” “Neutral,” “Agree,” and “Strongly Agree,” which correspond to scores of 1, 2, 3, 4, and 5, respectively.

### fMRI experimental procedures

The obtained speech video stimuli were presented using Psychtoolbox^25^. Videos were presented by a liquid crystal display (LCD) projector on a rear-projection screen mounted in the back of the scanner bore and were viewed through a mirror mounted to the head coil. The audio was played through MRI-compatible insert earphones. Participants were instructed to keep their heads still and attentively watch the videos. Before the task, they were told that they would need to answer a two-option question relevant to the speech content after each speech video (see Table 3). These questions were designed to ensure their engagement during video playing. Whole brain fMRI data were recorded while the participants were watching the speech videos. The order of the two videos was balanced among participants. After the scan, participants reported their experiences during the scanning (e.g., falling asleep and overall feeling of the speeches).

**Table 3:** Comprehension Questions for the HSS and the LSS.

Speech	Question	Options
**HSS**	The speaker thinks that art is ().	A. For me (correct answer) B. For you
**LSS**	The speaker’s attitude towards the emergence of many exaggerated designs in the current fashion industry is ().	A. Positive B. Negative (correct answer)

### fMRI data acquisition

Imaging data including T1-weighted structural and functional scans that were collected using the Siemens MAGNETOM Prisma 3 T MRI scanner using a 20-channel coil. High-resolution T1-weighted magnetization prepared rapid gradient echo sequences were first obtained, covering the whole brain with the following parameters: 208 slices, voxel size = 0.90 × 0.90 × 0.90 mm^3^, echo time = 2.32 ms, repetition time = 2300 ms, slice thickness = 0.9 mm, inversion time = 900 ms, flip angle = 8°, and the field of view = 240 × 240 mm^2^. Then, during each speech watching, fMRI sequences were collected by gradient echo planar imaging sequence with multi-band acceleration (repetition time = 1000 ms, echo time = 34 ms, voxel size = 2.50 × 2.50 × 2.50 mm^3^, voxel matrix = 92 × 92, flip angle = 50°, field of view = 230 × 230 mm^2^, slices number = 52, multiband-factor = 4).

#### fMRI data preprocessing

Raw DICOM files were sorted by sequence and converted to NIfTI format, and were organized according to the Brain Imaging Directory Structure (BIDS)^26^. All T1w images were defaced through *pydeface* toolbox (https://github.com/poldracklab/pydeface) for participants’ privacy and data security. Reports of image quality metrics were computed by MRIQC v21.0.0rc2 (https://github.com/nipreps/mriqc/)^27^. For estimation of framewise displacement of fMRI signal, processed fMRI signal was obtained through fMRIPrep pipeline^28^.

During the estimation of ISC, those fMRI images were preprocessed by the Data Processing & Analysis for Brain Imaging (DPABI, http://rfmri.org/DPABI)^29^. Preprocessing steps including (1) raw DICOM data were converted to NIFTI format, (2) slice-timing corrected and head motion correction with rigid body translation and rotation parameters, (3) co-registrating functional data to the Montreal Neurological Institute (MNI) space via T1 image unified segmentation, (4) removing the signal trend with time linearly, (5) regressing out nuisance signals including white matter signal, cerebrospinal fluid signal, global signal and Friston-24 head motion parameters. Then, (6) to reduce computational complexity, images were resampled into 3 × 3 × 3 mm3 isotropic voxel size. (7) applying a temporal band-pass filtering (0.01–0.1 Hz) and (8) spatial smoothing (a 6 mm full-width half-maximum (FWHM) Gaussian kernel was applied). The preprocessed images of each participant were used for the following ISC analyses.

## Data Records

The data collection using the BIDS data representation is available on OpenNeuro (https://openneuro.org/datasets/ds005920)^24^. All facial features have been removed from the structural and functional images. Our data include the raw fMRI data collected for each participant in the “sub-*” folders. Each participant folder contains two subfolders (see Table 4 for more information of the participants and data collection), named “anat” and “func”. The “anat” folder contains the T1 MRI data. The “func” folder contains two functional MRI data obtained when participants watched two speech videos. The *json* files contain information about the acquisition parameters. Related behavioral data and transcripts with timestamps for the two videos are available via a Figshare repository^30^. The original video files cannot be shared due to copyright restrictions, but the video files used in the experiment can be accessed via the links provided in the manuscript.

**Table 4:** Information of the Participants and Data Collection. Notes: "-" indicates that the data were not collected for that entry.

ID	Gender	Age	Run 1	Run 2	Notes
**sub-001**	male	22	HSS	LSS	
**sub-002**	male	22	LSS	HSS	
**sub-003**	male	24	HSS	LSS	
**sub-004**	male	20	HSS	LSS	
**sub-005**	female	20	LSS	HSS	Exclusion: incomplete data (technical issue)
**sub-006**	female	22	HSS	LSS	Exclusion: silent stimulus presentation
**sub-007**	female	20	LSS	HSS	Exclusion: silent stimulus presentation
**sub-008**	male	22	HSS	LSS	
**sub-009**	male	22	HSS	LSS	
**sub-010**	female	23	HSS	LSS	
**sub-011**	female	26	LSS	HSS	
**sub-012**	male	21	LSS	HSS	
**sub-013**	female	19	HSS	LSS	
**sub-014**	male	20	LSS	-	Exclusion: incomplete data (technical issue)
**sub-015**	male	19	LSS	HSS	Exclusion: silent stimulus presentation
**sub-016**	male	20	HSS	LSS	
**sub-017**	female	23	LSS	HSS	
**sub-018**	female	27	LSS	HSS	
**sub-019**	male	25	HSS	LSS	
**sub-020**	female	29	LSS	HSS	Exclusion: silent stimulus presentation
**sub-021**	male	31	LSS	HSS	
**sub-022**	female	23	HSS	LSS	
**sub-023**	female	23	LSS	HSS	
**sub-024**	male	23	LSS	HSS	
**sub-025**	female	20	LSS	HSS	
**sub-026**	female	20	HSS	LSS	
**sub-027**	male	20	HSS	LSS	
**sub-028**	male	20	HSS	-	Exclusion: incomplete data (late arrival)
**sub-029**	male	22	HSS	LSS	Exclusion: silent stimulus presentation
**sub-030**	female	22	HSS	LSS	
**sub-031**	male	21	LSS	HSS	Exclusion: sleep during scanning

## Technical Validation

To ensure the quality of the fMRI dataset, we conducted a comprehensive validation process, involving behavioral data analysis, head motion assessment, and inter-subject correlation (ISC) analysis. These validation steps were implemented to confirm that the data are robust and suitable for subsequent analyses, facilitating a detailed investigation of the neural mechanisms underlying audience engagement during public speaking.

### Quality control

**Fig. 3:**
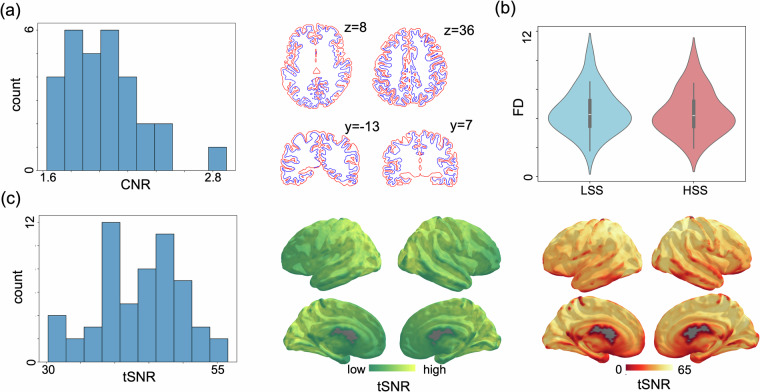
Image quality metrics across speeches. (**a**) Left panel: Contrast-to-noise (CNR) estimated in T1w scan, right panel: segmentation of grey and white matter for a participant. (**b**) Average framewise displacement (FD) of functional scans for each subject and each speech. No difference was observed between two speeches. (**c**) tSNR of functional scans. Left panel: The average tSNR was obtained for each speech (left panel), Middle panel: An exemplar individual's voxel-wise tSNR map, Right panel: The group-average voxel-wise tSNR across all participants.

The consistency of T1w scan quality was assessed using contrast-to-noise estimates computed in MRIQC. This metric provides a measure of separability of grey and white matter distributions for a given T1w image^27^, with higher values indicating better image quality (Fig. 3a).

For fMRI signal, brain-averaged framewise displacement (FD) was estimated, however, no difference was found between two scans for same participants (pair*-t* = 0.366, *p* = 0.717, Fig. 3b). Given the extended duration of the scanning sessions (approximately one hour), head motion was closely monitored and corrected during the preprocessing of fMRI data to ensure data quality. Framewise displacement (FD) was calculated for each participant to assess the extent of head movement. The analysis revealed that head motion was within acceptable limits for all participants, with no exclusions based on excessive movement. Unlike conventional exclusion criteria (e.g., mean framewise displacement [FD] exceeding 0.2 mm or more than 10% of volumes exceeding 0.5 mm FD), our approach did not apply strict thresholds due to the acceptable range of motion observed across all participants. Further, motion and distortion corrected time-series were used to calculate signal-to-noise (tSNR), which was calculated for each participant by dividing cortex-mapped timeseries by their standard deviation for each participant. Individual tSNR was averaged across the brain cortex, and voxel-wise tSNR values were averaged across subjects (Fig. 3c).

### Behavioral data analysis

To verify that participants were attentive during the fMRI scanning duration; a content-related question was administered following each speech video (see fMRI Experimental Procedure). These questions were intentionally designed to be straightforward, allowing participants who were attentive to answer correctly with ease. The results showed that nearly all participants answered both questions correctly for both the higher-scoring speech (HSS, accuracy: 100.0%) and the lower-scoring speech (LSS, accuracy: 83.9%), indicating that participants were attentive during the video presentations. This behavioral check serves as an initial validation of participant compliance and attentiveness, thereby supporting the reliability of the fMRI data.

### ISC analysis

Inter-subject correlation (ISC) analysis was employed to validate the dataset’s capacity to capture synchronized neural responses across participants. ISC reflects the degree to which the brain activity of multiple individuals becomes synchronized during a given task, indicating shared attention, understanding, and engagement. For each participant, we calculated the ISC of each voxel across the whole brain. The pair-wise ISC for each voxel was defined as Pearson’s correlation between the fMRI time-series of a pair of participants. For each participant, ISC at the individual level was obtained by averaging their pair-wise ISCs with all other participants. For visualization, brain maps of ISC at the individual level were then averaged across participants to establish ISC maps at the group level for both the HSS and LSS conditions, respectively.

**Fig. 4:**
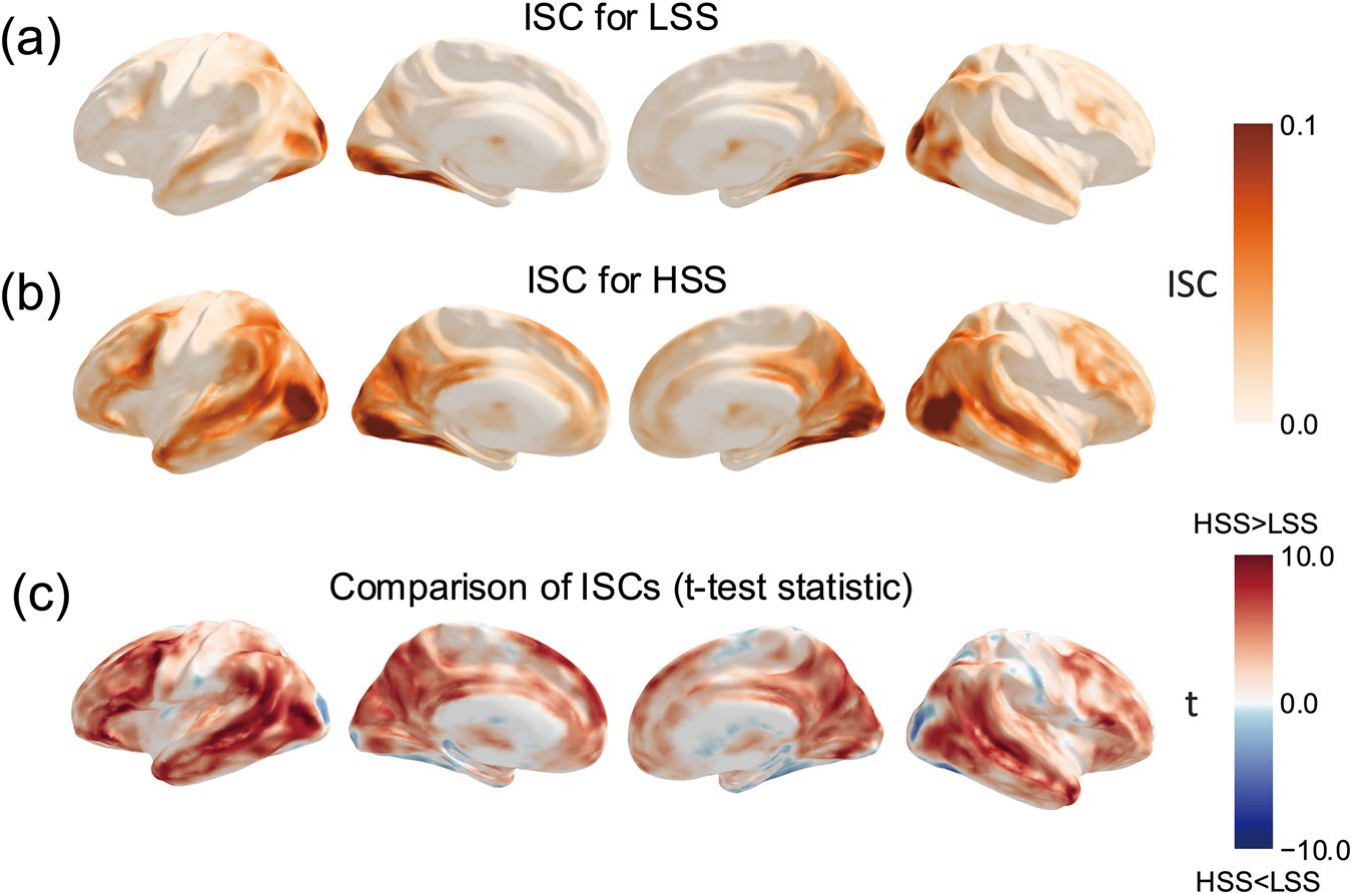
ISC maps and their comparison. (**a**) The ISC map for LSS. (**b**) The ISC map for HSS. (c) Statistical map showing the results of the voxel-wise t-test comparing ISC between LSS and HSS.

The resulting group-level ISC maps (see Fig. 4a,b) revealed significant neural synchronization in brain regions associated with visual and auditory perception, language processing, and social cognition, including the inferior frontal gyrus, superior temporal gyrus, and posterior cingulate cortex. A comparison of ISC results between the HSS and LSS conditions (see Fig. 4c) demonstrated greater synchronization for the HSS, suggesting that the more engaging speech evoked reliably coupled neural responses across listeners to a larger extent. These findings affirm the robustness of the fMRI data for investigating neural responses to public speaking and underscore the dataset’s potential for various analytical approaches.

## Usage Notes

This dataset offers a valuable resource for researchers across various fields, including cognitive and social neuroscience, communication studies, and educational science. While it was primarily designed for ISC analysis, the data is well-suited to address a broad range of research questions related to public speaking and prolonged brain activity dynamics. Future research could also explore temporal dynamics at a finer scale, such as using sliding-window approaches to capture how neural synchronization or functional connectivity pattern evolves over the course of a speech, identifying moments of peak engagement or disengagement. This temporal analysis could help identify critical segments within a speech that resonate most with the audience, providing practical implications for crafting more effective public communication.

Furthermore, comparing informative public speaking to teaching scenarios could offer valuable insights into the similarities and differences in neural mechanisms underlying these forms of knowledge transfer. Both contexts involve the transmission of complex information from a professional to a novice audience, but they differ in their interactive dynamics and goals. Teaching often involves bidirectional communication, feedback, and adaptive strategies to enhance understanding and retention, while public speaking typically follows a more unidirectional format. Future studies could utilize this dataset to compare neural responses in informative public speaking with data from classroom settings, examining whether similar brain networks are activated during information delivery or whether unique neural patterns emerge in response to interactive teaching elements.

While this dataset offers valuable possibilities, several limitations should be acknowledged. The restricted number of speech stimuli—one with a relatively higher engagement score and one with a lower engagement score—may limit the generalizability of the findings. This constraint arises from potential differences in variables beyond audience engagement, such as speaker loudness and video quality. Although we carefully selected and matched the stimuli to minimize these confounding factors, residual differences may still exist. Importantly, the observed functional relevance with specific brain networks associated with language and theory of mind largely mitigates these concerns. Nonetheless, future research should incorporate a broader range of materials when resources permit.

## Supplementary information

Table S1

## Data Availability

The code for ISC analysis is available on GitHub at the following link: https://github.com/cognomicslab/naturalistic_stimuli.
