# Supplementary material for: A naturalistic fMRI dataset in response to public speaking
Source: Sci Data. 2025 Apr 19;12:659. doi: 10.1038/s41597-025-05017-5 (PMC12009387; doi:10.1038/s41597-025-05017-5)
Supplement: Supplementary file 1 — Table S1 [file 41597_2025_5017_MOESM1_ESM.doc]

Table S1, 30 candidate speech videos and detailed information

| Number | Title | Time | Topic | Duration | Link |
| --- | --- | --- | --- | --- | --- |
| 1 | What are we living for | 2022.07.16 | Literature | 28'54'' | [https://www.yixi.tv/#/speech/detail?id=1103](https://www.yixi.tv/#/speech/detail?id=1103) |
| 2 | How to approach love in the golden age of singleness | 2017.06.10 | Love | 31'58'' | [https://www.yixi.tv/#/speech/detail?id=338](https://www.yixi.tv/#/speech/detail?id=338) |
| 3 | A long-term visual investigator | 2018.08.26 | Photography | 27'21'' | [https://www.yixi.tv/#/speech/detail?id=685](https://www.yixi.tv/#/speech/detail?id=685) |
| 4 | The prism of solitude | 2018.08.26 | Literature | 31'18'' | [https://www.yixi.tv/#/speech/detail?id=688](https://www.yixi.tv/#/speech/detail?id=688) |
| 5 | The truth that comes with time | 2019.10.27 | Literature | 30'02'' | [https://www.yixi.tv/#/speech/detail?id=832](https://www.yixi.tv/#/speech/detail?id=832) |
| 6 | This story is entirely non-fiction | 2016.12.22 | Life | 35'06'' | [https://www.yixi.tv/#/speech/detail?id=153](https://www.yixi.tv/#/speech/detail?id=153) |
| 7 | How can we draw a hedgehog? | 2019.12.28 | Nature | 35'14'' | [https://www.yixi.tv/#/speech/detail?id=868](https://www.yixi.tv/#/speech/detail?id=868) |
| 8 | What a more complete healthcare system looks like | 2023.01.08 | Health | 32'22'' | [https://www.yixi.tv/#/speech/detail?id=1149](https://www.yixi.tv/#/speech/detail?id=1149) |
| 9 | What is science | 2017.03.19 | Science | 29'20'' | [https://www.yixi.tv/#/speech/detail?id=144](https://www.yixi.tv/#/speech/detail?id=144) |
| 10 | What's wrong with our design | 2018.04.15 | Design | 24'13'' | [https://www.yixi.tv/#/speech/detail?id=646](https://www.yixi.tv/#/speech/detail?id=646) |
| 11 | Thank you for giving me the chance to take the stage | 2014.09.07 | Life | 34'46'' | [https://www.yixi.tv/#/speech/detail?id=114](https://www.yixi.tv/#/speech/detail?id=114) |
| 12 | How Bill Gates spends his money | 2018.07.15 | Society | 30'07'' | [https://www.yixi.tv/#/speech/detail?id=780](https://www.yixi.tv/#/speech/detail?id=780) |
| 13 | Cordelia's fate | 2017.10.22 | Literature | 27'19'' | [https://www.yixi.tv/#/speech/detail?id=586](https://www.yixi.tv/#/speech/detail?id=586) |
| 14 | What reading really is | 2016.11.27 | Culture | 22'18'' | [https://www.yixi.tv/#/speech/detail?id=298](https://www.yixi.tv/#/speech/detail?id=298) |
| 15 | The psychology behind anti-human-centered design | 2018.12.15 | Psychology | 26'59'' | [https://www.yixi.tv/#/speech/detail?id=741](https://www.yixi.tv/#/speech/detail?id=741) |
| 16 | Material heroes | 2019.12.28 | Design | 26'32'' | [https://www.yixi.tv/#/speech/detail?id=864](https://www.yixi.tv/#/speech/detail?id=864) |
| 17 | The urban tracker | 2018.03.11 | City | 36'47'' | [https://www.yixi.tv/#/speech/detail?id=620](https://www.yixi.tv/#/speech/detail?id=620) |
| 18 | The importance of salt reduction | 2019.10.27 | Health | 26'00'' | [https://www.yixi.tv/#/speech/detail?id=841](https://www.yixi.tv/#/speech/detail?id=841) |
| 19 | The prince and the ruins | 2022.09.25 | Literature | 26'02'' | [https://www.yixi.tv/#/speech/detail?id=1127](https://www.yixi.tv/#/speech/detail?id=1127) |
| 20 | Urban breathing | 2016.10.30 | Architecture | 25'55'' | [https://www.yixi.tv/#/speech/detail?id=120](https://www.yixi.tv/#/speech/detail?id=120) |
| 21 | Moss never disappears | 2017.08.19 | Literature | 24'39'' | [https://www.yixi.tv/#/speech/detail?id=579](https://www.yixi.tv/#/speech/detail?id=579) |
| 22 | Clothes, emotions, and women | 2017.08.19 | Design | 20'40'' | [https://www.yixi.tv/#/speech/detail?id=560](https://www.yixi.tv/#/speech/detail?id=560) |
| 23 | The self-cultivation of a young wild spirit | 2015.09.20 | Nature | 31'51'' | [https://www.yixi.tv/#/speech/detail?id=185](https://www.yixi.tv/#/speech/detail?id=185) |
| 24 | Grateful to have met you | 2017.04.08 | Photography | 24'04'' | [https://www.yixi.tv/#/speech/detail?id=139](https://www.yixi.tv/#/speech/detail?id=139) |
| 25 | Epiphyllum bloom | 2016.12.22 | Film | 31'42'' | [https://www.yixi.tv/#/speech/detail?id=40](https://www.yixi.tv/#/speech/detail?id=40) |
| 26 | Mai Po | 2017.07.15 | Environment | 21'29'' | [https://www.yixi.tv/#/speech/detail?id=565](https://www.yixi.tv/#/speech/detail?id=565) |
| 27 | The comic sage | 2020.08.23 | Imagination | 31'13'' | [https://www.yixi.tv/#/speech/detail?id=906](https://www.yixi.tv/#/speech/detail?id=906) |
| 28 | Choices at Lashi Lake | 2017.04.08 | Anthropology | 31'31'' | [https://www.yixi.tv/#/speech/detail?id=122](https://www.yixi.tv/#/speech/detail?id=122) |
| 29 | Gazing at oneself with a curious mind | 2013.10.20 | Anthropology | 20'47'' | [https://www.yixi.tv/#/speech/detail?id=236](https://www.yixi.tv/#/speech/detail?id=236) |
| 30 | Bigger | 2016.10.30 | Design | 24'28'' | [https://www.yixi.tv/#/speech/detail?id=303](https://www.yixi.tv/#/speech/detail?id=303) |
